# Children's Country Holidays Fund

**Published:** 1917-08-04

**Authors:** James Crichton-Browne

**Affiliations:** 18 Buckingham Street, Strand, W.C.


*To the Editor of* THE HOSPITAL.

### Children's Country Holidays Fund.

*To the Editor of* THE HOSPITAL.

SIR,—We are straining every nerve to save the babies, but the children are in need of salvation too, and one way of saving our town-bred and town-pent children is to provide for them a breath of fresh air and a glimpse of the land they live in. This the Children's Country Holidays Fund does, and I would earnestly plead for liberal support for it at a time when there is a special and urgent call for the tonic it supplies.

The great nervous upheaval caused by the war has not left the children unmoved. They reflect the feelings of their elders, and are involved in the chill and the shadow that have fallen on so many humble homes. Then, shell shock is not confined to the combatants. The air-raids have dazed some delicate children, and the rumours of war that circulate so ceaselessly around us have perturbed many more, stirring up the combative instincts of some, but startling and frightening others.

But contact with Nature and change of scene are eminently steadying and soothing in nervous disturbance, and these the Holidays Fund offers to our war-worn children, while to all children, whether war-worn or not, it affords educational influences that are priceless. Many of us are no doubt curtailing our wonted holiday at this strenuous time, but the children should have theirs in ampler measure than before, and it is to be hoped that this truly benign charity, the Children's Country Holidays Fund, will be enabled to extend its wholesome and joy-giving usefulness. Donations should be sent to the Secretary, C.C.H.F., 18 Buckingham Street, Strand, W.C. 2.—Your obedient servant,

JAMES CRICHTON-BROWNE.

18 Buckingham Street, Strand, W.C., July 28, 1917.

